# Impact of e-ASPECTS software on the performance of physicians compared to a consensus ground truth: a multi-reader, multi-case study

**DOI:** 10.3389/fneur.2023.1221255

**Published:** 2023-09-07

**Authors:** Hassan Kobeissi, David F. Kallmes, John Benson, Alex Nagelschneider, Ajay Madhavan, Steven A. Messina, Kara Schwartz, Norbert Campeau, Carrie M. Carr, Deena M. Nasr, Sherri Braksick, Eugene L. Scharf, James Klaas, Zoe Victoria Joan Woodhead, George Harston, James Briggs, Olivier Joly, Stephen Gerry, Anna L. Kuhn, Angelos A. Kostas, Kambiz Nael, Mohamad AbdalKader, Ramanathan Kadirvel, Waleed Brinjikji

**Affiliations:** ^1^Department of Radiology, Mayo Clinic, Rochester, MN, United States; ^2^Department of Neurology, Mayo Clinic, Rochester, MN, United States; ^3^Brainomix Limited, Oxford, United Kingdom; ^4^Acute Stroke Service, Oxford University Hospitals NHSFT, Oxford, United Kingdom; ^5^Royal Berkshire NHS Foundation Trust, Reading, United Kingdom; ^6^Centre for Statistics in Medicine, University of Oxford, Oxford, United Kingdom; ^7^Division of Neurointerventional Radiology, Department of Radiology, UMass Medical Center, Worcester, MA, United States; ^8^Huntington Hospital and Hill Medical Imaging, Pasadena, CA, United States; ^9^Department of Radiological Sciences, David Geffen School of Medicine, University of California, Los Angeles (UCLA), Los Angeles, CA, United States; ^10^Department of Radiology, Boston Medical Center, Boston, MA, United States; ^11^Department of Neurologic Surgery, Mayo Clinic, Rochester, MN, United States

**Keywords:** imaging, stroke, ASPECTS, neuroradiology, thrombectomy

## Abstract

**Background:**

The Alberta Stroke Program Early CT Score **(**ASPECTS) is used to quantify the extent of injury to the brain following acute ischemic stroke (AIS) and to inform treatment decisions. The e-ASPECTS software uses artificial intelligence methods to automatically process non-contrast CT (NCCT) brain scans from patients with AIS affecting the middle cerebral artery (MCA) territory and generate an ASPECTS. This study aimed to evaluate the impact of e-ASPECTS (Brainomix, Oxford, UK) on the performance of US physicians compared to a consensus ground truth.

**Methods:**

The study used a multi-reader, multi-case design. A total of 10 US board-certified physicians (neurologists and neuroradiologists) scored 54 NCCT brain scans of patients with AIS affecting the MCA territory. Each reader scored each scan on two occasions: once with and once without reference to the e-ASPECTS software, in random order. Agreement with a reference standard (expert consensus read with reference to follow-up imaging) was evaluated with and without software support.

**Results:**

A comparison of the area under the curve (AUC) for each reader showed a significant improvement from 0.81 to 0.83 (*p* = 0.028) with the support of the e-ASPECTS tool. The agreement of reader ASPECTS scoring with the reference standard was improved with e-ASPECTS compared to unassisted reading of scans: Cohen's kappa improved from 0.60 to 0.65, and the case-based weighted Kappa improved from 0.70 to 0.81.

**Conclusion:**

Decision support with the e-ASPECTS software significantly improves the accuracy of ASPECTS scoring, even by expert US neurologists and neuroradiologists.

## Introduction

The Alberta Stroke Program Early CT Score (ASPECTS) is an 11-point scale (0–10) with which the extent of ischemic change in the middle cerebral artery (MCA) territory of acute ischemic stroke (AIS) can be quantified on a non-contrast CT (NCCT) scan of the brain (1, 2). Guidelines in both the United States and Europe recommend using ASPECTS alongside clinical and other imaging criteria to guide patient selection for reperfusion therapy treatment in AIS (3, 4). The European guidelines also cite the use of ASPECTS to help guide treatment decisions for thrombolysis in the context of severe stroke (5). However, numerous studies have shown inconsistent scoring of ASPECTS, even by trained raters (6, 7).

Considering the inconsistent scoring between readers, automated decision support software, such as e-ASPECTS (Brainomix, Oxford, UK), has been developed to facilitate consistent evaluation of the NCCT head scan and improve accuracy. Standalone studies have validated the accuracy of the e-ASPECTS software in the setting of AIS in the MCA territory, both with reference to the ASPECTS (8–13), and the volume generated by the heatmap (14–16), which underlies the e-ASPECTS output. Independent studies have also been shown to improve the ASPECTS assessment of scans when using the e-ASPECTS software compared with not using this decision support (17).

In this study, the impact of e-ASPECTS decision support software was quantified for a group of 10 US board-certified neurologists and neuroradiologists. The readers were randomly allocated e-ASPECTS decision support for two reading sessions at least a month apart.

## Methods

### Patients and scan acquisition

A total of 54 patient scans were acquired from a cohort registry of patients with confirmed AIS of the MCA territory presenting to the Mayo Clinic, Rochester, United States, between July 2015 and February 2020. Patient eligibility for endovascular therapy was determined per the institutional protocol at the time of presentation. Cases not meeting the study criteria were not included. The diagnosis was confirmed by the treating team with reference to clinical and comprehensive imaging data (including CT angiography to confirm the vascular territory of the stroke).

Serial patients meeting the following criteria were included in this study: AIS affecting the MCA territory; eligible for acute endovascular reperfusion therapy; no evidence of intracranial hemorrhage; imaging data of adequate quality (e.g., free from excessive motion leading to major artifacts) with associated demographic and follow-up imaging available.

All imaging was acquired on a SIEMENS scanner (SOMATOM Definition Flash 39/Edge 15), with NCCT imaging available in appropriate reconstructions. For reader purposes, the presented data were 3–5 mm reconstructions to maximize the pathological contrast to noise ratio.

### Ground truth determination (reference standard)

A consensus of three board-certified neuroradiologists (for whom ASPECTS scoring on the NCCT is part of their clinical practice) was used as the reference standard for analysis (9, 11). Each expert was given a demonstration of how to use the scoring platform and provided with training material. The three neuroradiologists independently scored each of the 54 CT scans with reference to the clinical information provided (including laterality and severity of symptoms and treatment success) and follow-up clinical imaging. To enhance the accuracy of the ground truth, the truthers were given additional clinical information and follow-up imaging to facilitate their reads. The ground truth was established by the consensus of the three expert neuroradiologist readers for each region scored, and when there was no complete consensus, the region was attributed to the status of most readers.

### Reader task

The 10 clinician readers comprised representative intended e-ASPECTS users from the United States, including neurologists and neuroradiologists, all of whom interpret NCCT scans as part of their clinical routine. The readers consisted of four neurologists and six neuroradiologists, all with US board certification. The time post-board certification varied from <1 year to 22 years, with a median of 6 years. All readers used the same viewing platform, with and without e-ASPECTS support.

Each reader scored each ASPECTS region of every scan in two sessions, at least 4 weeks apart. The readers were given only the acute NCCT image for their scoring. In the first session, half of the cases were selected at random to be presented with e-ASPECTS decision support. The other half of the cases were presented without e-ASPECTS decision support. In the second session, the decision support allocation was reversed.

The reader indicated which hemisphere was affected, and within the hemisphere, which of the ASPECTS regions was affected. Scans scored with reference to the e-ASPECTS overlay were presented alongside the non-overlayed scan using the standard e-ASPECTS interface, which is the same format as that output to a standard hospital imaging platform (PACS). Scans read without e-ASPECTS decision support were presented without reference to the e-ASPECTS region segmentation framework to best represent standard clinical practice.

### Statistical analysis

The primary endpoint was the diagnostic accuracy of each rater compared to the reference standard (consensus read defined by the ground truthers), quantified using the area under the curve (AUC) of the receiver operating characteristic (ROC). This provides a composite measure of the impact on both sensitivity and specificity. The sample size calculation was derived based on the unified Obuchowski and Rockette–Dorman, Berbaum, and Metz (OR–DBM) analysis methods devised by Hillis, Obuchowski, and Berbaum (18). The sample size was powered to detect a difference in AUC of 0.095 (0.1 × 0.95). With an alpha of 0.05, the sample size calculation indicated that 54 patient cases and 10 readers would allow a power of 80%.

Secondary endpoints included sensitivity, specificity, overall percentage agreement (accuracy), and Cohen's Kappa statistic. Bland–Altman analysis was used to evaluate the impact on magnitude of the variation in agreement with the reference standard. Bias was defined as the mean difference between the reader's score with and without e-ASPECTS. An analysis of agreement at the patient level was described using a weighted Kappa statistic. Statistics were reported with reference to upper and lower 95% confidence interval limits. Statistical significance was determined by a *p* < 0.05.

## Results

### Patient characteristics

A total of 54 representative NCCT brain scans were included in this study. Demographic data for the patients included are described in Table 1. The median age was 72 years, and the median time of imaging from the last known well was 3 h 19 min, reflective of the intended use of ASPECTS in patients presenting within the time window for reperfusion therapies (4).

**Table 1:** Characteristics of the included patients.

**Patient characteristic**	**Result (*n =* 54)**
Age [median (IQR)]	72 (57–79)
Female	26 (48%)
NIHSS at presentation to hospital [median (IQR)]	17 (11–21)
Right hemisphere	21 (39%)
Proximal occlusion	40 (74%)
Time from known symptom onset to scan, hh:mm [median (range)]	03:19 (00:52–12:41)
Number of patients presenting >6 h from onset	10 (19%)
Unknown symptom onset time	8 (15%)

NIHSS, National Institutes of Health Stroke Scale; IQR, interquartile range. Proximal occlusion defined as internal carotid artery or middle cerebral artery M1.

### Imaging characteristics

Imaging characteristics were described using the consensus of the ground truthers, including total ASPECTS and the presence of commonly encountered co-existing imaging findings. The distribution of ASPECTS (Supplementary Table S1) observed in the unenriched study cohort is reflective of the population for whom use is intended. The observed median ASPECTS of 9 is consistent with the figures reported within a large meta-analysis of patients undergoing assessment of MCA territory AIS (19).

In all, 20 (37%) patients were noted to have incidental chronic white matter disease, and 12 (22%) patients had non-acute incidental infarcts visible on the CT scan. The prevalence of these coincident findings is comparable to those seen in cohorts described in the stroke literature (20). One patient was noted to have an incidental meningioma, which did not impact the image processing.

Comparison of the AUC for each reader with and without the support of the e-ASPECTS tool showed an improvement of 0.02 from 0.81 (95% confidence interval [CI] 0.76–0.86) to 0.83 (95% CI 0.79–0.87; difference = 0.02, 95% CI 0.00–0.04; *p* = 0.028) (Table 2).

**Table 2:** Overall study results outlining the accuracy compared to the reference standard with e-ASPECTS decision support compared to unassisted reads (95% confidence interval in brackets).

	**AUC**	**Specificity**	**Sensitivity**	**Accuracy**	**Cohen's Kappa**	**Weighted Kappa**
Without	0.81 (0.76, 0.86)	0.96 (0.94, 0.97)	0.66 (0.56, 0.76)	0.93 (0.91, 0.94)	0.60 (0.54, 0.65)	0.70 (0.61, 0.79)
With	0.83 (0.79, 0.87)	0.96 (0.95, 0.97)	0.70 (0.61, 0.79)	0.94 (0.92, 0.95)	0.65 (0.59, 0.71)	0.81 (0.75, 0.87)
Difference	0.02 (0.00, 0.04)	0.01 (0.00, 0.01)	0.04 (0.00, 0.09)	0.01 (0.00, 0.02)	0.05 (0.02, 0.10)	0.11 (0.06, 0.016)
*p*-value	0.028	0.005	0.100	0.012	0.013	<0.001

AUC, area under the curve.

### Evaluation of ASPECTS reads

When comparing reader performance with the ground truth, the improved AUC in the primary endpoint was driven by an increase in both sensitivity and specificity when assisted by e-ASPECTS compared to unassisted reading (see Table 2 for quantitative results). Overall percentage agreement (accuracy) also improved. Agreement of ASPECTS scoring with the reference standard was improved with e-ASPECTS compared to unassisted reading of scans: Cohen's kappa improved significantly from 0.60 to 0.65 (*p* = 0.013) and the case-based weighted Kappa improved significantly from 0.70 to 0.81 (*p* < 0.001) (Table 2).

**Figure 1:**
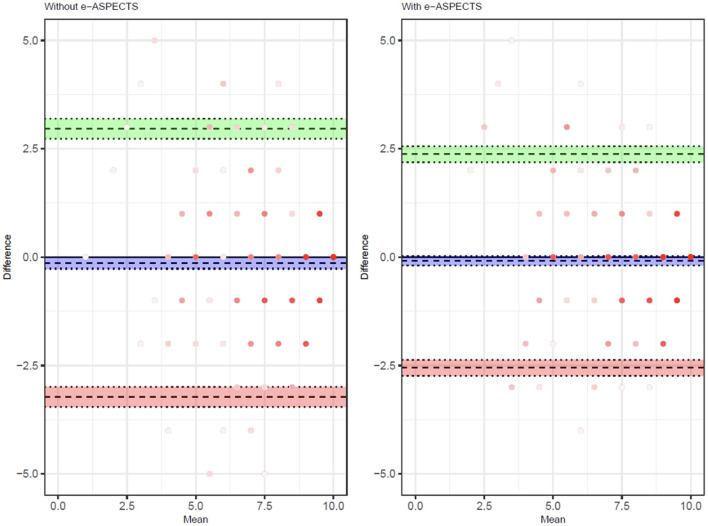
Bland–Altman showing the distribution of reader scores compared to the ground truth unassisted **(left)** and with e-ASPECTS support **(right)**.

The improvement in the overall level of accuracy is demonstrated in the Bland–Altmann plot (Figure 1), showing a reduction in the limits of agreement of total ASPECTS and a reduction in bias (Supplementary Table S2). A subgroup analysis based on the clinical training of the reader (radiologist vs. neurologist) demonstrates a consistent impact of e-ASPECTS across reader groups (Supplementary Table S3).

**Figure 2:**
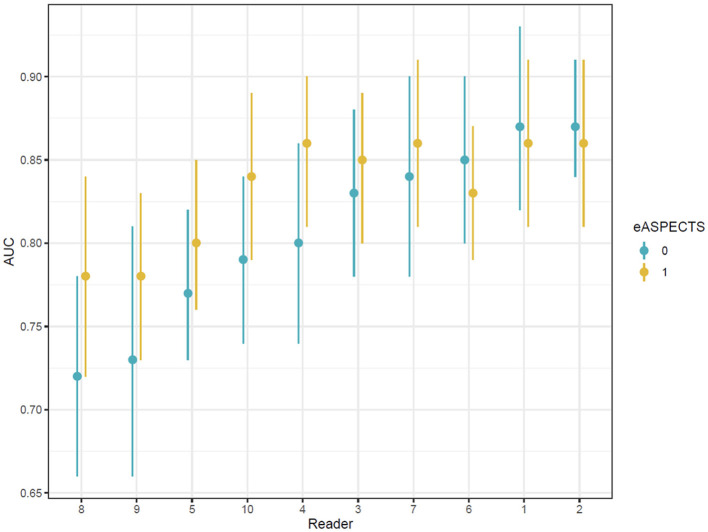
Area under the ROC curve values for each reader ordered according to baseline performance.

Analysis of individual reader ROC curves showed a greater magnitude of increases in AUC observed in users with lower initial performances and smaller changes in readers with higher unassisted performance. The range in AUC between users was also narrower with e-ASPECTS than unassisted (Supplementary Figure S1 and Figure 2), indicating a reduction in the variation of performance between different readers when e-ASPECTS outputs are available. Similarly, the Bland–Altman plots for individual readers also show greater consistency with the reference standard with e-ASPECTS at an individual level (Supplementary Figure S2).

### Subgroup analyses

To further explore the generalizability of the impact of e-ASPECTS, subgroup analyses were undertaken to investigate the performance of e-ASPECTS in deep vs. cortical regions in patients with high vs. low ASPECTS. The use of e-ASPECTS improved reader performance in both the deep (lentiform, caudate, and internal capsule) and cortical (insula and M1–M6) regions (Supplementary Table S4).

Furthermore, the benefit of e-ASPECTS was shown to be consistent across subgroups with low ASPECTS (≤ 6; *N* = 12) and high ASPECTS (>6; *N* = 42; see Supplementary Table S5).

## Discussion

This study demonstrates the ability of an FDA-cleared artificial intelligence (AI) decision support tool (e-ASPECTS) to improve the performance of US physicians when deriving the ASPECTS. Consistent improvement was seen in both neurology and neuroradiology-qualified doctors.

Although there are a significant number of published studies describing the standalone performance of the AI software compared to a reference ground truth (9, 12, 13, 21–23), there is less data regarding the impact of the AI software on physician performance. Previous studies that have examined this impact on reader ASPECTS scoring performance have used a reference standard defined by independent imaging modalities (17), non-US physicians (24), or physicians who are less specialized and for whom it is easier to show an impact (25).

Brinjikji et al. (17) reported a reader study with 60 cases and 16 readers in which the ground truth was established using the consensus view of two readers using follow-up (24 h) CT or MRI ASPECTS. A potential criticism of this approach is that the ischemic area may change between baseline and follow-up imaging. The study showed an intraclass correlation coefficient of 0.395 for unassisted readers (without e-ASPECTS) and 0.574 for assisted readers (with e-ASPECTS); this improvement was statistically significant.

Scavasine et al. (24) previously reported a reader study with 116 cases and four Brazilian readers (two neuroradiologists and two emergency physicians). This study also used follow-up imaging along with clinical information to ascertain the ground truth. The results showed that the performance of the emergency physicians improved with the use of e-ASPECTS and neared that of neuroradiologists.

Delio et al. (25) reported a reader study using a similar software device. The study used 50 cases and 8 readers (2 neuroradiologists and 6 non-neuroradiologists), with the ground truth established by the consensus of three expert neuroradiologists with access to acute and follow-up imaging. The results showed that the percentage agreement between readers and ground truth improved from 72.4% (unassisted) to 77.9% (assisted) on average for the non-neuroradiologists; however, there was no change for the expert neuroradiologists.

In contrast to previous studies, this study used a consensus of neuroradiologists with access to clinical and follow-up data to define the ground truth, and the positive impact of e-ASPECTS was demonstrated for both neurologists and expert neuroradiologists. To the best of our knowledge, this is the first time a benefit has been shown to neuroradiologists using a consensus ground truth.

This study evaluated the impact of e-ASPECTS on both patients and readers, which is representative of a high standard of care in the United States. Importantly, the readers whose performance was evaluated were all board-certified physicians specialized in stroke care from a leading US institution. The demographics of the patient population were comparable to those seen in prospective stroke trials that have informed guidelines and led to the widespread use of the ASPECTS methodology in clinical practice (26). Co-existing findings related to patient co-morbidity (including white matter disease and old infarcts) were also representative of those seen in clinical practice.

The improvement in accuracy between scans read with e-ASPECTS and those without e-ASPECTS in this study was driven by both improvements in sensitivity and specificity using a region-based analysis. This also resulted in more consistent scoring and greater agreement at the patient level, as reflected by more accurate overall scores. The improved performance was consistent between both radiology-trained and non-radiology-trained doctors, which may reflect the comparable expertise for those components of image interpretation such as ASPECTS that are used for treatment decisions.

The subgroup analysis demonstrated a consistent impact of e-ASPECTS on reader performance in deep and superficial regions. Subgrouping by total ASPECTS (≤ 6 and >6) showed that the impact of the device on reader performance is greatest in the low ASPECTS subgroup. Interestingly, the improvement in the low ASPECTS subgroup is driven by improved sensitivity to ischemia (and reduced under-calling of abnormalities), where there are more abnormal regions resulting in lower ASPECTS. In the high ASPECTS subgroup, the effect is mainly on improved specificity of reads. This leads to no overall bias on total scores, but a better discrimination of high vs. low ASPECTS dichotomized around 6. The Bland-Altman analysis of the total ASPECTS demonstrates that there is no systematic bias in e-ASPECTS performance in aided vs. unaided reads, and there is no trend in bias due to the total ASPECTS. Although we found that results differed by scored region, these results should be further investigated in subsequent, higher-powered studies.

This study has several limitations. First, the use of expert readers means that any impact of e-ASPECTS is likely to underestimate that seen by readers at less specialist hospitals. Second, the ground truth was set by expert consensus with reference to clinical data and follow-up imaging. Despite having access to this additional information, ASPECTS is known to vary between even expert readers, and so although this provides a reference standard, it cannot be considered an absolute truth (27). Alternative reference standards would be required to demonstrate this, such as MRI imaging at follow-up in patients who have had early and complete recanalization. Our study did not consider the time from onset in the analysis. Future studies should further explore the impact of e-ASPECTS in different treatment windows.

## Conclusion

The results of this study indicate that decision support with the e-ASPECTS software significantly improves the assessment of ASPECTS scoring by expert US physicians and reduces variation in assessment between readers.

## Data availability statement

The raw data supporting the conclusions of this article will be made available by the authors, without undue reservation.

## Ethics statement

The studies involving humans were approved by the Mayo Clinic Institutional Review Board. The studies were conducted in accordance with the local legislation and institutional requirements. Written informed consent for participation was not required from the participants or the participants' legal guardians/next of kin in accordance with the national legislation and institutional requirements.

## Author contributions

WB is the overall guarantor of the study. All authors contributed to the drafting of the manuscript.
