# Supplementary material for: Impact of e-ASPECTS software on the performance of physicians compared to a consensus ground truth: a multi-reader, multi-case study
Source: Front Neurol. 2023 Sep 7;14:1221255. doi: 10.3389/fneur.2023.1221255 (PMC10513025; doi:10.3389/fneur.2023.1221255)
Supplement: Supplementary file 1 [file Data_Sheet_1.docx]

**
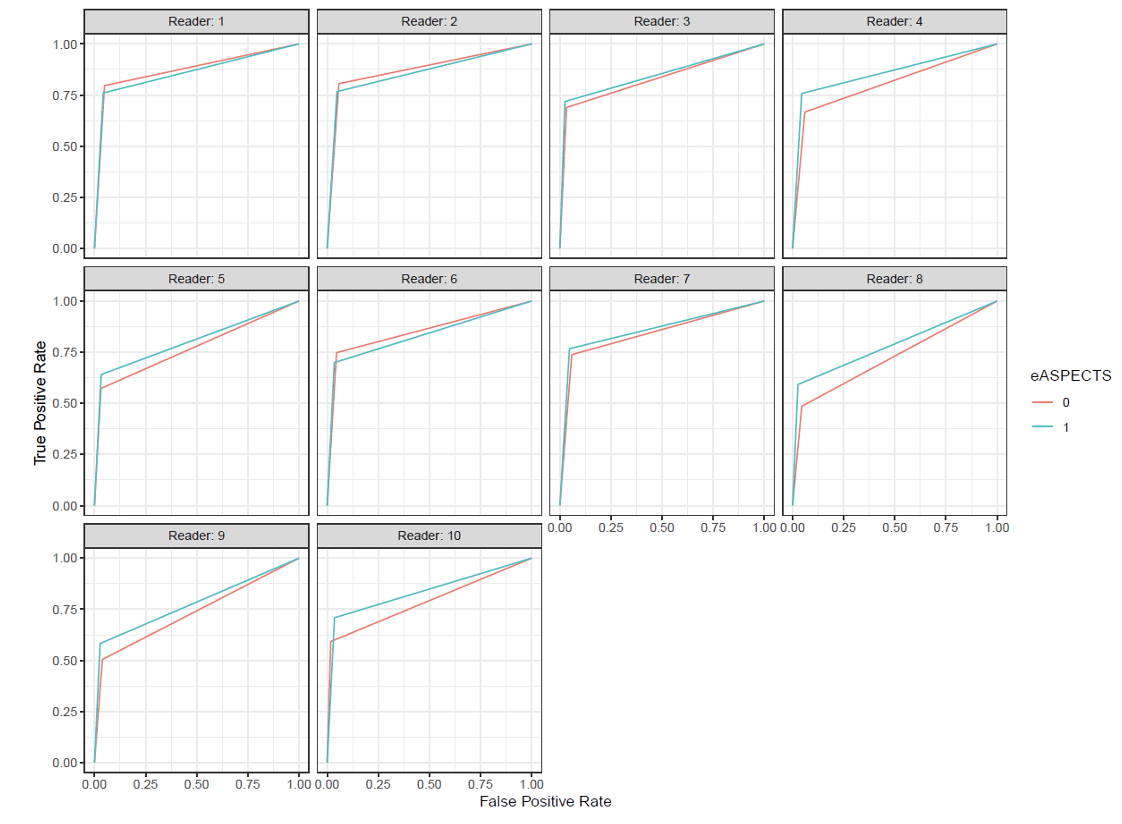
**

**Supplementary Figure S1.** ROC curve analysis for individual readers unassisted (eASPECTS=0) and with e-ASPECTS (eASPECTS=1) support.

**
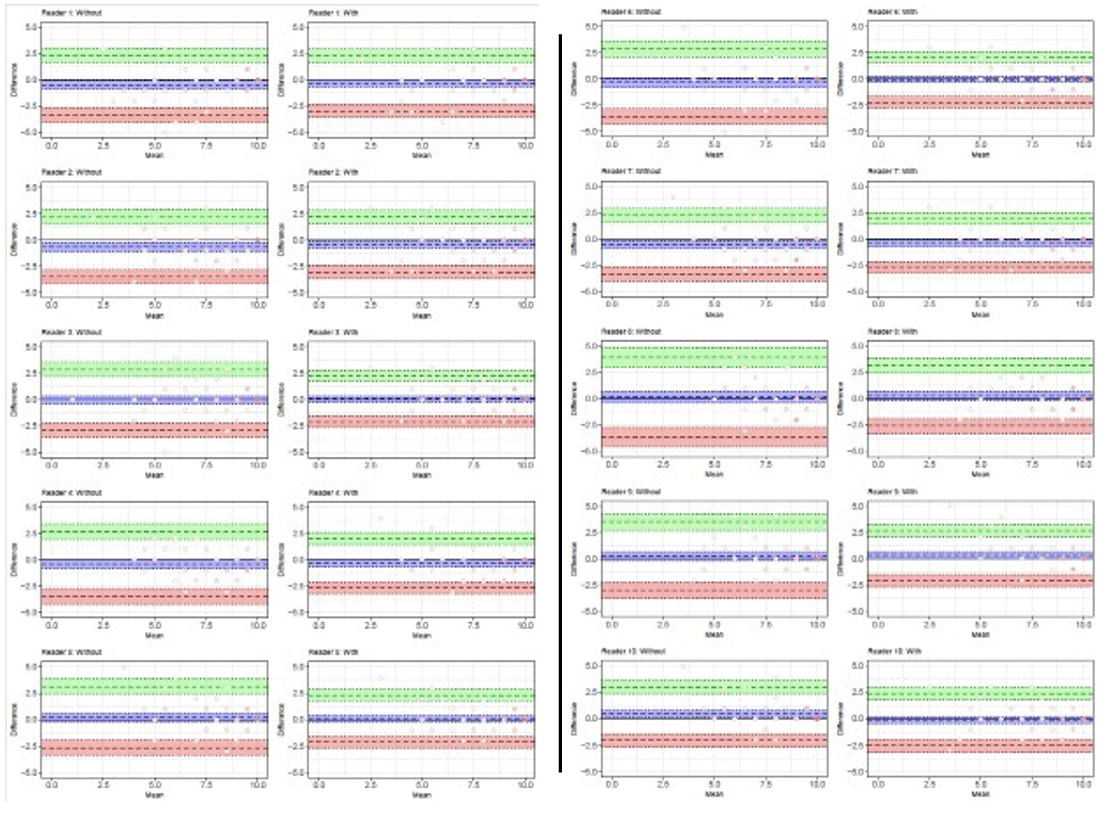
**

**Supplementary Figure S2.** Bland-Altman plots showing the distribution of individual reader scores compared to the ground truth unassisted (left panels) and with e-ASPECTS support (right panels). The reader scores become closer to the ground truth with use of e-ASPECTS.

**Supplementary Table S1. Distribution of ASPECTS in the cohort according to ground truth data***.*

| **ASPECTS score** | **Number** |
| --- | --- |
| 9-10 | 31 (57%) |
| 6-8 | 14 (26%) |
| <6 | 9 (17%) |

**Supplementary Table S2. Bias and limits of agreement from the Bland-Altman analysis displayed in Figure 1**

|  | Bias | Upper limit of agreement | Lower limit of agreement |
| --- | --- | --- | --- |
| Without | -0.13 (-0.27, 0.00) | 2.96  (2.73, 3.19) | -3.23 (-3.46, -3.00) |
| With | -0.09  (-0.19, 0.02) | 2.38  (2.19, 2.56) | -2.55  (-2.73, -2.37) |

**Supplementary Table S3. Study results by reader background outlining the accuracy compared to the reference consensus with e-ASPECTS decision support compared to unassisted reads**

| **Reader Specialty** |  | **AUC** | **Specificity** | **Sensitivity** | **Accuracy** | **Cohen’s Kappa** | **Weighted Kappa** |
| --- | --- | --- | --- | --- | --- | --- | --- |
| **Neuroradiologist** | Without | 0.82 (0.77, 0.87) | 0.96 (0.94, 0.98) | 0.69 (0.57, 0.80) | 0.93 (0.91, 0.95) | 0.63 (0.57, 0.68) | 0.74 (0.65, 0.82) |
|  | With | 0.84 (0.80, 0.88) | 0.96 (0.95, 0.97) | 0.73 (0.64, 0.81) | 0.94 (0.92, 0.95) | 0.66 (0.61, 0.72) | 0.82 (0.74, 0.87) |
|  | Difference | 0.02 (-0.01, 0.05) | 0.00 (-0.01, 0.02) | 0.04 (-0.03, 0.11) | 0.01 (0.00, 0.01) | 0.04 (0.00, 0.07) | 0.08 (0.04, 0.12) |
| **Neurologist** | Without | 0.79 (0.69, 0.89) | 0.95 (0.94, 0.96) | 0.62 (0.41, 0.83) | 0.92 (0.89, 0.94) | 0.56 (0.48, 0.62) | 0.65 (0.48, 0.75) |
|  | With | 0.81 (0.75, 0.87) | 0.97 (0.95, 0.98) | 0.66 (0.53, 0.79) | 0.94 (0.91, 0.95) | 0.63 (0.56, 0.69) | 0.79 (0.72, 0.85) |
|  | Difference | 0.03 (-0.02, 0.07) | 0.01 (0.01, 0.02) | 0.04 (-0.06, 0.14) | 0.02 (0.01, 0.03) | 0.08 (0.02, 0.15) | 0.15 (0.07, 0.26) |

**Supplementary Table S4. Study results in cortical (M1-M6 and insula) versus deep (lentiform, caudate, internal capsule) regions outlining the accuracy with e-ASPECTS decision support compared to unassisted reads.**

| **Region Group** |  |  | **AUC** | **Specificity** | **Sensitivity** | **Accuracy** | **Cohen’s Kappa** |
| --- | --- | --- | --- | --- | --- | --- | --- |
| M1-M6, Insula |  | Without | 0.80 (0.76, 0.85) | 0.96 (0.95, 0.97) | 0.65 (0.55, 0.75) | 0.93 (0.92, 0.95) | 0.60 (0.54, 0.66) |
|  |  | With | 0.83 (0.78, 0.88) | 0.97 (0.96, 0.98) | 0.69 (0.60, 0.78) | 0.95 (0.92, 0.96) | 0.67 (0.59, 0.74) |
|  |  | Difference | 0.03 (-0.01, 0.06) | 0.01 (0.00, 0.02) | 0.04 (-0.02, 0.11) | 0.01 (0.00, 0.02) | 0.06 (0.01, 0.11) |
| Lentiform, Caudate, Internal Capsule |  | Without | 0.81 (0.73, 0.89) | 0.94 (0.92, 0.96) | 0.68 (0.52, 0.85) | 0.91 (0.88, 0.93) | 0.59 (0.48, 0.67) |
|  |  | With | 0.83 (0.76, 0.91) | 0.94 (0.92, 0.96) | 0.72 (0.57, 0.87) | 0.92 (0.89, 0.94) | 0.62 (0.52, 0.71) |
|  |  | Difference | 0.02 (-0.02, 0.06) | 0.00 (-0.01, 0.02) | 0.04 (-0.03, 0.11) | 0.01 (0.00, 0.02) | 0.03 (-0.03, 0.07) |

**Supplementary Table S5. Study results in patients with low (<=6) or high (>6) ASPECTS.**

| **ASPECTS** | |  | | **AUC** | | **Specificity** | | **Sensitivity** | | **Accuracy** | | **Cohen’s Kappa** | | **Weighted Kappa** | |
| --- | --- | --- | --- | --- | --- | --- | --- | --- | --- | --- | --- | --- | --- | --- | --- |
| Low, ≤6  (N=12) | | Without | | 0.80 (0.75, 0.85) | | 0.94 (0.92, 0.96) | | 0.66 (0.53, 0.78) | | 0.87 (0.82, 0.88) | | 0.63 (0.55, 0.69) | |  | |
|  |  | With | | 0.83 (0.77, 0.88) | | 0.94 (0.91, 0.97) | | 0.72 (0.60, 0.83) | | 0.88 (0.83, 0.91) | | 0.68 (0.59, 0.75) | |  | |
|  |  | Difference | | 0.03 (-0.01, 0.06) | | 0.00 (-0.03, 0.02) | | 0.06 (-0.01, 0.13) | | 0.01 (0.00, 0.04) | | 0.04 (-0.01, 0.12) | |  | |
|  |  |  | |  | |  | |  | |  | |  | |  | |
| High, >6  (N=42) | | Without | | 0.81 (0.76, 0.88) | | 0.96 (0.95, 0.97) | | 0.66 (0.52, 0.80) | | 0.95 (0.93, 0.96) | | 0.51 (0.43, 0.60) | |  | |
|  |  | With | | 0.82 (0.76, 0.88) | | 0.97 (0.96, 0.98) | | 0.67 (0.54, 0.80) | | 0.96 (0.94, 0.96) | | 0.56 (0.46, 0.64) | |  | |
|  |  | Difference | | 0.01 (-0.03, 0.05) | | 0.01 (0.00, 0.02) | | 0.01 (-0.07, 0.09) | | 0.01 (0.00, 0.02) | | 0.05 (-0.02, 0.10) | |  | |
|  |  |  | |  | |  | |  | |  | |  | |  | |
